# Case Report: Vancomycin-Associated Tubulointerstitial Nephritis in Clinical Practice-Case Report and Review of Literature

**DOI:** 10.3389/fmed.2022.899886

**Published:** 2022-05-31

**Authors:** Lakshmi Kannan, Rishi Raj

**Affiliations:** ^1^Department of Internal Medicine, Division of Nephrology, Pikeville Medical Center, Pikeville, KY, United States; ^2^Department of Internal Medicine, Pikeville Medical Center, Pikeville, KY, United States

**Keywords:** vancomycin, acute interstitial nephritis, nephrotoxicity, MRSA, kidney biopsy

## Abstract

Nephrotoxicity is one of the major limiting factors for vancomycin use. The most common histological patterns of kidney injury are acute tubulointerstitial nephritis and acute tubular necrosis. Patients who develop acute tubulointerstitial nephritis are prone to develop acute kidney injury with vancomycin rechallenge and, in most cases, present alone or as a part of Drug Reaction with Eosinophilia and Systemic Symptoms (DRESS). The purpose of the review study is to identify biopsy-proven vancomycin-associated-tubulointerstitial nephritis in literature, determine possible underlying pathophysiology and identify the consequences of vancomycin rechallenge in such patients.

## Introduction

Vancomycin, famously known as “Mississippi Mud,” was first approved for use by the Food and Drug Administration in 1958 (1). It is the antibiotic of choice for gram-positive infections, mainly methicillin-resistant *Staphylococcus aureus* (MRSA). Despite significant nephrotoxicity associated with vancomycin, it still has widespread use, being the drug of choice in the emergency department/acute setting for initial broader coverage. Vancomycin-associated tubulointerstitial nephritis (VAIN) is a recognizable histopathological pattern observed in patients receiving vancomycin. We present a patient with biopsy proven vancomycin-associated tubulointerstitial nephritis who was re-introduced to the drug after a year of developing maculopapular rash and mild acute kidney injury. We identified 14 case reports with VAIN, nine of which were biopsy-proven. We found that the kidney injury was self-limiting and improved by discontinuing vancomycin; however, steroids were used in some cases. Only three studies showed rapid recurrence of maculopapular rash and/or acute oligo-anuric kidney injury with vancomycin rechallenge.

## Case Presentation

**FIGURE 1:**
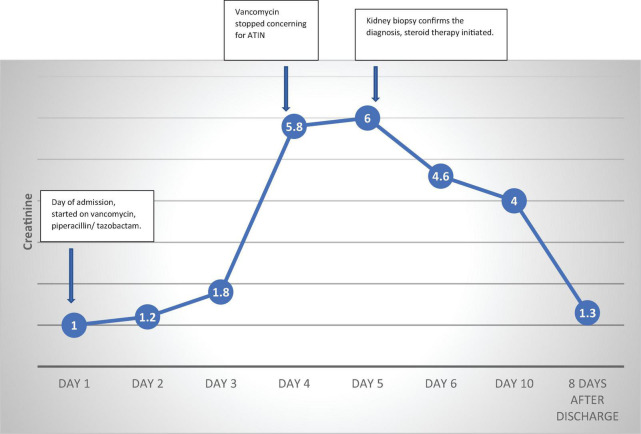
Creatinine trend. ATIN, Acute tubulointerstitial nephritis.

**FIGURE 2:**
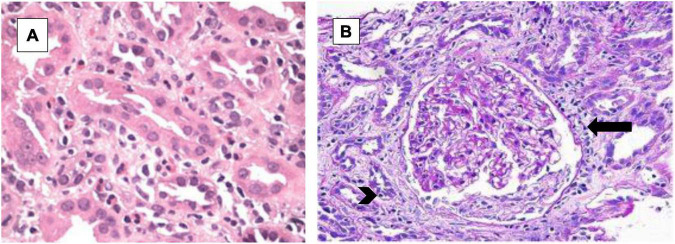
Hematoxylin and eosin (H&E) stain. **(A)** Low power- diffuse cellular infiltrate within the interstitium with inflammatory cells including eosinophils and lymphocytes. **(B)** High power- the tissue sample shows the presence of inflammatory cell infiltrate within the tubular wall (arrowhead) and numerous eosinophils in the interstitium (arrow).

We present the case of a 56-year-old man with hypertension, poorly controlled type II diabetes mellitus (DM) and hyperlipidemia who presented to the Emergency Room with generalized fatigue, fever and an open draining ulcer on his right heel. Laboratory work up showed elevated white blood cell count of 18.95 K/μL (3–11.3 K/μL), initial blood urea nitrogen of 18 mg/dL (6–24 mg/dL) and creatinine of 1.0 mg/dL (0.6–1.10 mg/dL) and rest of the blood work as shown in Table 1. His blood pressure was controlled with lisinopril 40 mg daily, hyperlipidemia with atorvastatin 40mg daily and his DM with insulin therapy. The patient was started on initial broad-spectrum antibiotics with vancomycin 1 g every 12 h (70-kilogram male) and piperacillin- tazobactam 4.5 g every 8 h after drawing blood cultures. On Hospital Day 1, his blood culture was positive for gram positive cocci, so Piperacillin was stopped. Two days later, he developed pitting pedal edema, reduced urine output (250 ml/day) and serum creatinine increased up to 5.8 mg/dL as shown in Table 1 and creatinine trend shown in Figure 1. His vancomycin trough was 17 mcg/ml. Urinalysis showed moderate hematuria with 10–20 red blood cells and 3 + protein. Microscopic examination of the sediment showed a single white blood cell cast. Given concern for acute tubulointerstitial nephritis, vancomycin was immediately stopped, kidney biopsy was obtained which confirmed the presence of eosinophils in the interstitium and interstitial cells within the tubules as shown in Figure 2. After this event, his previous hospital records were reviewed and it was discovered that the patient was hospitalized a year back with a diabetic foot ulcer and at that time, he had received vancomycin which resulted in him developing maculopapular rash and mild acute kidney injury. Vancomycin was stopped due to concern for an allergic reaction and his symptoms had resolved with a short course of prednisone (500 mg of intravenous methylprednisolone for 3 days followed by oral prednisone at 1 mg/kg tapered over 14 days).

**TABLE 1:** Laboratory work-up at the time of admission and on hospital day 4.

Laboratory test	Reference range	At the time of admission	Hospital day 4
Hemoglobin	11–17 mg/dL	10.1	10
Hematocrit	32–51.6%	31	31
White blood cells	3–11.3 K/μL	18.95	16
Platelets	134–412 K/μL	304	288
Serum glucose	70–110 mg/dL	116	98
Serum sodium	133–144 mmol/L	138	135
Serum potassium	3.6–5.2 mmol/L	4	3.8
Serum calcium	8.3–10.4 mg/dL	9.6	9.4
Serum magnesium	1.7–2.4 mg/dL	2.1	2.0
Serum bicarbonate	21–32 mmol/L	23	24
Serum phosphorus	3.5–5.0 mg/dL	4.2	4.0
Blood urea nitrogen	6–24 ng/dL	18	65
Serum creatinine	0.60–1.10 mg/dL	1.0	5.8
Estimated glomerular filtration rate	(> 60 mL/min/1.73m2)	48	18
Serum albumin	3.4–5.4 mg/dL	2.9	2.6
Serum aspartate transaminases	15–37 U/L	15	20
Serum alanine transaminases	12–78 U/L	18	22
Serum alkaline phosphatase	44–147 U/L	146	145
Hemoglobin A1C	<6.5%	7.1	NA
Urine microalbumin, random	1.3–30 mg/dL	74	164
Erythrocyte sedimentation rate	(0–20 mm/hr)	78	NA
C-reactive protein	(0–0.3 mg/dL)	14	NA
Urine protein creatinine ratio	<0.2	0.7	1.2
Vancomycin trough	(5–15 mcg/ml)	NA	17

*NA, Not available.*

## Discussion

One of the limiting factors of vancomycin use is kidney injury. Vancomycin related acute kidney injury (AKI) is defined as at least two or three consecutive elevations in serum creatinine by 0.5 mg/dl or at least a 50% increase from the baseline, whichever is greater; the increase must be documented after several days of vancomycin therapy, and no alternative explanation for the impairment in glomerular filtration rate is identified (2).

Vancomycin can cause varied immune-mediated reactions or hypersensitivity reactions (HSR)-immediate, type I IgE mediated HSR, type II antibody-mediated HSR, and type IV cell-mediated delayed HSR. These HSR include Drug Reaction with Eosinophilia and Systemic Symptoms (DRESS), IgA bullous dermatosis, and Steven-Johnson Syndrome/Toxic Epidermal Necrolysis (SJS/TEN). Acute tubulointerstitial nephritis (AIN) is a part of type II antibody-mediated HSR.

The first case of vancomycin-associated tubulointerstitial nephritis was published in 1981 (3), but it was not until 1988 that the first biopsy-proven acute tubulointerstitial nephritis was described (4) as shown in Table 2. Histologically, vancomycin-associated tubulointerstitial nephritis is identified by the presence of features like tubulitis (57.9%), followed by eosinophilic infiltration (42.1%), epithelial sloughing (42.1%), tubular casts (42.1%), and interstitial fibrosis (36.8%) (5).

**TABLE 2:** A Literature review of vancomycin-associated tubulointerstitial nephritis.

Author, year	Indication for vancomycin use	Suspicion for AIN	Kidney biopsy	Other variables	Re-challenge
Eisenberg et al. (3)	Right-sided endocarditis and septic emboli	AKI, sterile pyuria, eosinophiluria.	No	Gentamycin and indomethacin	ND
Michail et al. (4)	Staphylococcal chest infection, MRSA loculated pleural effusion.	AKI and Henoch-Schoenlein purpura	Tubulo-interstitial nephritis	None	ND
Codding et al. (8)	*Staphylococcus aureus* endocarditis	Maculopapular rash, anuric AKI.	No	None	Re-challenge several days later resulted in reappearance of rash and anuric AKI.
Azar et al. (9)	Enterococcus endocarditis	Rapid onset of oligo-anuric AKI.	Tubulo-interstitial nephritis	None	Rechallenge after several days- reappearance of rash and acute oligo-anuric AKI.
Wai et al. (10)	*Staphylococcus aureus* sternal wound, Osteomyelitis, and IE.	Fever, maculopapular rash, eosinophilia, eosinophiluria.	Tubulo-interstitial nephritis	Cloxacillin and Ciprofloxacin	4 months after initial episode, rechallenged for septic arthritis and developed eosinophilia and eosinophiluria.
Hsu et al. (11)	Iliopsoas MRSA abscess	Rash, eosinophilia, AKI	Diffuse and marked interstitial and tubular infiltration by mononuclear cells and eosinophils	Oxacillin, ceftriaxone, amikacin, rifampicin, gentamycin, and Bactrim	ND
Zuliani et al. (12)	MRSA	DRESS, AIN	No	None	ND
Hong et al. (13)	Gram positive bacteremia	AKI	Granulomatous interstitial nephritis.	Lisinopril and ibuprofen.	ND
Plakogianniset al. (14)	Suspected bacterial meningitis	AKI	No, creatinine improved with discontinuation of medications.	Ceftriaxone and acyclovir.	ND
Salazar et al. (15)	MRSA osteomyelitis	Rash, AKI	No, kidney function improved with stopping vancomycin and starting steroids.	Gentamycin	ND
Htike et al. (16)	Coagulase-negative staphylococcus bacteremia	AKI	Acute interstitial nephritis with lymphocytic and eosinophilic infiltrate and acute tubular necrosis	None	ND
Diaz- mancebo et al. (17)	MRSA cervical spondylodiscitis	Rash, fever, Oliguria followed by anuria	Diffuse moderate tubulointerstitial lesion is detected, with inflammatory infiltrates made up of polymorphs formed by small lymphocytes, plasma cells and abundant eosinophils	Piperacillin- tazobactam	ND
Pingili et al. (18)	MRSA bacteremia	AKI, lower extremity edema, diffuse maculopapular rash.	Sclerosed glomeruli, some with mesangial proliferation, and tubulointerstitial inflammation with eosinophils and plasma cells and mild interstitial fibrosis.		ND
Swada et al. (19)	Fournier gangrene	AKI	Acute tubular necrosis and focal acute tubulointerstitial nephritis.		ND

*ATIN, Acute tubulointerstitial nephritis; AKI, Acute Kidney Injury; MRSA, Methicillin resistant Staphylococcus aureus; DRESS, Drug Reaction with Eosinophilia and Systemic Symptoms; IE, Infective Endocarditis.*

Another histological pattern seen with vancomycin is ATN- Animal studies demonstrated that vancomycin leads to tubular damage and interstitial inflammation related to oxidative stress in the proximal tubular cells and vasoconstriction by free oxygen radicals (6). It can also alter mitochondrial function. Oxidative phosphorylation by vancomycin induces free oxygen radicals, thereby reducing the activity of defensive antioxidative enzymes, including superoxide dismutase and catalases. Vancomycin superoxide production causes mitochondrial membrane potential depolarization with the release of cytochrome C and subsequent activation of caspases 9 and 3, leading to apoptotic cell death.

Vancomycin, in addition, causes immunological injury by acting as an exogenous antigen that can be directly trapped within the interstitium or circulate as an immune complex that gets deposited subsequently within the interstitium. Alternatively, it can bind to tubular antigens and act as haptens or perform molecular mimicry of the tubules and/or interstitium. Both cell-mediated and humoral immunity can be involved in the process, but it is initiated when an antigen-presenting lymphocyte is activated. This results in the formation of activated T-cells that promote differentiation and proliferation of other T-cells resulting in cytokine and chemokine production, including TGF-β, PDGF-β, EGF-β, and fibroblast growth factor-2. Over time, tubulointerstitial inflammation and edema transition to fibrosis and tubular loss (7).

In the case of vancomycin, the clinical presentation is quite variable. It comes to attention when there is an unexplained rise in serum creatinine after exposure to vancomycin. Nevertheless, the clinical picture is often complicated by multiple processes ongoing during hospitalization or otherwise, such as systemic infection, bouts of hemodynamic instability, and concomitant use of other nephrotoxic medications. Most often, the suspicion for vancomycin-associated tubulointerstitial nephritis is low as symptoms are non-specific and the classic triad of fever, rash, and eosinophilia suggestive of hypersensitivity are present only in a minority of patients (> 5–10%) (7).

Objectively, there is no one laboratory test that can be used to diagnose VAIN. Most commonly observed laboratory abnormalities are eosinophilia, increased serum IgE levels, elevated erythrocyte sedimentation rate, and anemia. Urinalysis can show hematuria, pyuria, and proteinuria on dipstick testing. Urine eosinophils are neither sensitive nor specific for the diagnosis of VAIN as it is seen in cystitis or prostatitis, pyelonephritis, atheroembolic disease, acute tubular necrosis, and rapidly progressive glomerulonephritis. White blood cell casts seen on urine microscopy in a patient with AKI without pyelonephritis are highly suggestive of VAIN.

Since vancomycin-associated tubulointerstitial nephritis represents a hypersensitivity reaction, sometimes with systemic symptoms and dermatological manifestations, a kidney biopsy can provide diagnostic information regarding exact etiology, decision toward discontinuation of the drug, corticosteroid administration, and prediction of long-term prognosis. Nonetheless, it is often difficult to establish causality due to confounders/risk factors as seen from the literature review (including presence of other antibiotics, non-steroidal anti-inflammatory drugs, severe sepsis) and biopsy timing in relation to the use of vancomycin. In patients who cannot undergo kidney biopsy, scanning with 67Ga scintigraphy or positron emission tomography scanning may be utilized to differentiate VAIN from acute tubular necrosis (ATN) (20).

## Conclusion

Vancomycin-associated tubulointerstitial nephritis is presumed to be more frequent than we encounter in clinical practice, and hence the reported literature has been regarded as the tip of the iceberg. Survival analysis has linked the presence of acute tubulointerstitial nephritis to a worse prognosis. Hence, performing a timely kidney biopsy is paramount to diagnosing and treating tubulointerstitial nephritis. Furthermore, for biopsy-proven AIN, future rechallenges with vancomycin should be avoided, and such patients should be listed as having had an adverse reaction to vancomycin.

## Data Availability Statement

The original contributions presented in the study are included in the article/supplementary material, further inquiries can be directed to the corresponding author.

## Ethics Statement

Ethical review and approval was not required for the study on human participants in accordance with the local legislation and institutional requirements. The patients/participants provided their written informed consent to participate in this study. Written informed consent was obtained from the individual(s) for the publication of any potentially identifiable images or data included in this article.

## Author Contributions

LK: conception, design of the study, review of literature, and analysis of data. RR: drafting of the manuscript and review of the final manuscript. Both authors contributed to the article and approved the submitted version.

## Conflict of Interest

The authors declare that the research was conducted in the absence of any commercial or financial relationships that could be construed as a potential conflict of interest.

## Publisher’s Note

All claims expressed in this article are solely those of the authors and do not necessarily represent those of their affiliated organizations, or those of the publisher, the editors and the reviewers. Any product that may be evaluated in this article, or claim that may be made by its manufacturer, is not guaranteed or endorsed by the publisher.

## References

[1] Levine DP. Vancomycin: a history. *Clin Infect Dis.* (2006) 42:S5–12. 10.1086/491709 16323120

[2] Rybak M Lomaestro B Rotschafer JC Moellering R Craig W Billeter M Therapeutic monitoring of vancomycin in adult patients: a consensus review of the American Society of Health-System Pharmacists, the Infectious Diseases Society of America, and the Society of Infectious Diseases Pharmacists. *Am J Health Syst Pharm.* (2009) 66:82–98. 10.2146/ajhp080434 19106348

[3] Eisenberg ES Robbins N Lenci M. Vancomycin and Interstitial Nephritis. *Ann Intern Med.* (1981) 95:658. 10.7326/0003-4819-95-5-658_1 7294568

[4] Michail S Vaiopoulos G Nakopoulou L Revenas C Aroni K Karam P Henoch-schoenlein purpura and acute interstitial nephritis after intravenous vancomycin administration in a patient with a staphylococcal infection: CASE REPORT. *Scand J Rheumatol.* (1998) 27:233–5. 10.1080/030097498440886 9645421

[5] Bellos I Pergialiotis V Perrea DN. Kidney biopsy findings in vancomycin-induced acute kidney injury: a pooled analysis. *Int Urol Nephrol.* (2022) 54:137–48. 10.1007/s11255-021-02831-9 33715061

[6] Bamgbola O. Review of vancomycin-induced renal toxicity: an update. *Ther Adv Endocrinol Metab.* (2016) 7:136–47. 10.1177/2042018816638223 27293542 PMC4892398

[7] Perazella MA. AKI in a Hospitalized Patient with Cellulitis. *Clin J Am Soc Nephrol.* (2013) 8:658–64. 10.2215/CJN.09370912 23099655

[8] Codding CE Ramseyer L Allon M Pitha J Rodriguez M. Tubulointerstitial nephritis due to vancomycin. *Am J Kidney Dis.* (1989) 14:512–5. 10.1016/S0272-6386(89)80152-0 2596477

[9] Azar R Bakhache E Boldron A. [Acute interstitial nephropathy induced by vancomycin]. *Nephrologie.* (1996) 17:327–8. 8975151

[10] Wai AO Lo AM Abdo A Marra F. Vancomycin-Induced Acute Interstitial Nephritis. *Ann Pharmacother.* (1998) 32:1160–4. 10.1345/aph.17448 9825081

[11] Hong Hsu SI. Biopsy-Proved Acute Tubulointerstitial Nephritis and Toxic Epidermal Necrolysis Associated with Vancomycin. *Pharmacotherapy.* (2001) 21:1233–9. 10.1592/phco.21.15.1233.33901 11601669

[12] Zuliani E Zwahlen H Gilliet F Marone C. Vancomycin-induced hypersensitivity reaction with acute renal failure: resolution following cyclosporine treatment. *Clin Nephrol.* (2005) 64:155–8. 10.5414/CNP64155 16114793

[13] Hong S Valderrama E Mattana J Shah HH Wagner JD Esposito M Vancomycin-induced acute granulomatous interstitial nephritis: therapeutic options. *Am J Med Sci.* (2007) 334:296–300. 10.1097/MAJ.0b013e3180a6ec1e 18030187

[14] Plakogiannis R Nogid A. Acute interstitial nephritis associated with coadministration of vancomycin and ceftriaxone: case series and review of the literature. *Pharmacotherapy.* (2007) 27:1456–61. 10.1592/phco.27.10.1456 17896901

[15] Salazar MN Matthews M Posadas A Ehsan M Graeber C. Biopsy proven interstitial nephritis following treatment with vancomycin: a case report. *Conn Med.* (2010) 74:139–41. 20391819

[16] Htike NL Santoro J Gilbert B Elfenbein IB Teehan G. Biopsy-proven vancomycin-associated interstitial nephritis and acute tubular necrosis. *Clin Exp Nephrol.* (2012) 16:320–4. 10.1007/s10157-011-0559-1 22086124

[17] Díaz-Mancebo R Costero-Fernández O Vega-Cabrera C Olea-Tejero T Yébenes L Picazo ML Dress syndrome and acute tubulointerstitial nephritis after treatment with vancomycin and beta-lactams. Case report and literature review. *Nefrologia.* (2012) 32:685–7. 10.3265/Nefrologia.pre2012.Jun.11455 23013963

[18] Pingili CS Okon EE. Vancomycin-induced leukocytoclastic vasculitis and acute renal failure due to tubulointerstitial nephritis. *Am J Case Rep.* (2017) 18:1024–7. 10.12659/AJCR.905214 28943633 PMC5627863

[19] Sawada A Kawanishi K Morikawa S Nakano T Kodama M Mitobe M Biopsy-proven vancomycin-induced acute kidney injury: a case report and literature review. *BMC Nephrol.* (2018) 19:72. 10.1186/s12882-018-0845-1 29587650 PMC5872390

[20] Perazella MA. Diagnosing drug-induced AIN in the hospitalized patient: A challenge for the clinician. *Clin Nephrol.* (2014) 81:381–8. 10.5414/CN108301 24691017 PMC4326856

